# Effect of Infliximab Dose Increase in Rheumatoid Arthritis at Different Trough Concentrations: A Cohort Study in Clinical Practice Conditions

**DOI:** 10.3389/fmed.2015.00071

**Published:** 2015-10-08

**Authors:** Chamaida Plasencia, Teresa Jurado, Alejandro Villalba, Diana Peitedado, Maria Teresa López Casla, Laura Nuño, María Gema Bonilla, Ana Martínez-Feito, Emilio Martín-Mola, Dora Pascual-Salcedo, Alejandro Balsa

**Affiliations:** ^1^Rheumatology Unit, Instituto de Investigación Sanitaria del Hospital Universitario La Paz (IdiPAZ), Madrid, Spain; ^2^Immunology Unit, Instituto de Investigación Sanitaria del Hospital Universitario La Paz (IdiPAZ), Madrid, Spain

**Keywords:** rheumatoid arthritis, infliximab, dose increase, clinical efficacy

## Abstract

**Background:**

Evidence supporting treatment intensification in rheumatoid arthritis (RA) is limited and controversial. We explored outcomes of infliximab dose increases and accounted for pre-existing trough levels in patients with active RA.

**Methods:**

This study was a retrospective study of 42 RA patients who received increased infliximab following an insufficient response (DAS28 >3.2). Serum concentrations of infliximab and antibodies to infliximab (ATI) and DAS28 and EULAR clinical response parameters were recorded for 1 year. Analyses were performed in three patient groups that were defined by infliximab serum concentration prior to treatment enhancement: no detectable, low (<1.1 μg/mL) or high (≥1.1 μg/mL) drug levels.

**Results:**

No circulating infliximab was detected in 20 patients (47.6%), but 13 (31%) and 9 (21.4%) patients exhibited low and high levels, respectively. ATI was only detected in patients with no detectable drug levels because the drug interferes with ELISA. DAS28 disease activity globally showed a modest improvement after dose escalation, but this improvement did not persist after 6 and 12 months. Infliximab serum levels increased significantly in the high group (*p* = 0.016), but no increase was achieved in the low and no detectable groups. The three study groups exhibited similar disease activity over time, and no improvement was observed in the non-responder EULAR rates.

**Conclusion:**

These results suggest that the efficacy of an infliximab dose increase is limited, and the response is independent of the infliximab trough serum concentration that is achieved prior to escalation.

## Introduction

The response to tumor necrosis factor (TNF) inhibitor treatment in chronic inflammatory diseases exhibits great therapeutic variability. Failure to respond to anti-TNF therapy may occur at treatment onset, or it may be secondary to an initial improvement (1, 2). A concentration-dependent effect was described for anti-TNF treatment (3, 4), and inadequate serum trough drug levels is a major cause for non-responsiveness (3). The development of anti-drug antibodies [antibodies to infliximab (ATI)] is a major source of drug clearance, and it is associated with lower serum drug levels and lack of response (5). However, an optimal therapeutic concentration is not defined, and empirical algorithms for treatment optimization prevail (6). A change in therapeutic target would be appropriate in non-improving patients with high circulating infliximab (Ifx) levels, and switching to another TNF inhibitor was proposed in patients who present no free drug levels and with detectable anti-drug antibodies following treatment. However, increasing the dose of anti-TNF treatment when drug levels are low may achieve a threshold therapeutic concentration (7, 8), and this approach was effective in inflammatory bowel disease (9).

Evidence supporting dose intensification in rheumatoid arthritis (RA) is limited and controversial, and few studies relate dose intensification with pre-increase serum trough drug levels (10–12).

The present study retrospectively analyzed the effect of Ifx dose increase in RA non-responders and accounted for previous drug concentrations to further elucidate the utility of proposed algorithms for patients who do not respond to the first anti-TNF therapy.

## Materials and Methods

A retrospective observational study was conducted of RA patients included in the Hospital Universitario La Paz Biologics Registry (Madrid, Spain) who started treatment with Ifx as the first TNF-blocking agent. The following inclusion criteria were used: RA patients (older than 18 years) treated from 2005 to 2011 who exhibited an insufficient clinical response defined as DAS28 >3.2, who received an increase in the dose of Ifx, and who had serum sample and clinical assessment data during the first year of treatment. The period of inclusion ended in 2011 because no increase in Ifx dose was used in our clinic after this time. A final observation was carried forward for analysis in patients who stopped Ifx treatment within the first year after dose increase.

Ifx treatment could be combined with classic disease-modifying anti-rheumatic drugs (DMARDs) and/or corticosteroids. The research ethics committee of the Hospital Universitario La Paz (Madrid, Spain) approved the study, and informed consent was obtained for the storage and future use of serum samples.

### Treatment

Patients were initially treated with 3 mg/kg Ifx intravenously at weeks 0, 2, 6, and 14 and every 8 weeks thereafter. The dose of Ifx was increased via the administration of 4, 5, or 6 mg/kg Ifx or a reduction in the administration interval to 7 or 6 weeks, with a maximum dose of 6 mg/kg every 6 weeks. Dose escalation could also be combined with increasing doses of DMARDs and/or corticosteroids.

### Data collection and assessments

Medical history, demographic data, anti-citrullinated protein antibodies (ACPA), and rheumatoid factor (RF) were retrospectively retrieved prior to Ifx increase (baseline). DAS28 score, EULAR response, and serum concentrations of Ifx and ATI were also retrieved at baseline (T1), after the first Ifx dose increment (T2), and at 6 months (T3), and 12 months (T4). ACPA were measured using ELISA (Eurodiagnostica, Malmo, Sweden), and RF was assessed using nephelometry (Siemens, Marburg, Germany) with cut-off values of 25 and 9 IU/mL. DAS28 was calculated using the erythrocyte sedimentation rate (ESR), and the response to Ifx was evaluated using the European League Against Rheumatism (EULAR) criteria (13).

### Infliximab serum and ATI concentrations

Serum Ifx concentrations were determined using a capture ELISA as described previously (14), but a biotinylated monoclonal anti-Ifx idiotype antibody (Progenika Bipopharma S.A., Vizcaya, Spain) was used instead of a rabbit antibody to detect Ifx. Serum ATI levels were assayed using a two-site (bridging) in-house ELISA (15) with a cut-off for positivity of 50 arbitrary units (AU)/mL.

### Statistical analysis

Descriptive statistics are provided as the mean, SD, median and interquartile range (IQR). A fixed effects analysis of repeated measures was performed, and Bonferroni correction was used for multiple comparisons. Qualitative variables were compared at different time points and between different groups using Fisher’s test, and the Bonferroni test was used for multiple comparisons.

A regression mixed model for repeated measurements was performed using group (no, low, and high IFX levels) and time points (T1, T2, T3, and T4) as factors to compare DAS28 and delta-DAS28 between groups. Interactions between factors were calculated as fixed effects, and subjects were calculated as random effects. Pair-wise comparisons were calculated using Bonferroni correction. EULAR was analyzed using a generalized linear model with cumulative logit link function. A significance level of 0.05 was used for statistical testing, and all analyses were performed using SAS 9.3 (SAS Institute, Cary, NC, USA).

Patients were assigned to one of three serum Ifx groups: no detectable, low (<1.1 μg/mL) or high (≥1.1 μg/mL). These cut-off levels were based on the observation that serum Ifx >1 mg/L (12) was associated with improved disease (16).

## Results

### Patient characteristics

Forty-two patients (37 women) were included in the study. Twenty patients exhibited no detectable free Ifx, 13 patients exhibited Low levels, and 9 patients exhibited High levels. Table 1 shows the descriptive statistics.

**Table 1:** **Demographic and clinical characteristics of patients**.

	Total study population (*n* = 42)	No detectable Ifx levels (*n* = 20)	Low Ifx levels (*n* = 13)	High Ifx levels (*n* = 9)
Age at onset (years), mean ± SD	57.1 ± 14.0	49.6 ± 14.5	61.6 ± 10.8	67.4 ± 6.1
Female, *n* (%)	37 (88.1)	19 (95)	9 (69.2)	9 (100)
Disease duration (years), mean ± SD	19.4 ± 10.4	16.3 ± 6.3	17.9 ± 10.1	28.3 ± 13.8
Duration of Ifx treatment (years), median (IQR)	6.2 (1–13)	4.25 (1.23–8.63)	6.25 (4.38–10.75)	8.25 (8.25–10.25)
ACPA-positive, *n* (%)	36 (85.7)	19 (95)	12 (92.3)	5 (55.6)
RF-positive, *n* (%)	35 (83.3)	18 (90)	10 (76.9)	7 (77.8)
Methotrexate therapy, *n* (%)	36 (85.7)	16 (80)	12 (92.3)	8 (88.8)
Methotrexate dose (mg/week), median (IQR)	12.5 (0–25)	15.0 (0–15)	15 (0–20)	10 (0–15)
Other DMARDs, *n* (%)	18 (42.9)	9 (21.4)	4 (9.2)	5 (11.9)
Concomitant use of glucocorticoids, *n* (%)	28 (66.6)	13 (30.9)	8 (19)	7 (16.6)
Prednisone dose (mg/day) before Ifx increase, mean ± SD	6.2 ± 5.2	5.7 ± 6.8	4.2 ± 3.2	7.0 ± 6.4
Prednisone dose (mg/day) at one year, mean ± SD	7.9 ± 6.3	8.7 ± 7.6	7.1 ± 6.3	8.2 ± 5.6
DAS28 at the start Ifx treatment, mean ± SD	5.50 ± 1.20	5.68 ± 1.29	5.03 ± 1.04	5.77 ± 1.12
Baseline DAS28 before Ifx increase, mean ± SD	4.55 ± 1.01	4.91 ± 0.73	3.72 ± 0.90	4.97 ± 1.06
Trough Ifx levels before dose increase (μg/mL), median (IQR)	94.5 (0–10.5)	ND	574 (16–1024)	2112 (1152–10464)
ATI levels before Ifx increase, AU/mL, median (IQR)	0 (0–60000)	1068.5 (377.5–12328.0)	0 (0–0)	ND

*Ifx, Infliximab; SD, standard deviation; IQR, interquartile range; ACPA, anti-citrullinated peptide antibodies; RF, rheumatoid factor; DMARDs, disease-modifying anti-rheumatic drugs; DAS28, disease activity score in 28 joints; ATI, anti-infliximab antibodies; AU/mL, arbitrary units per milliliter; ND, not detectable*.

The age of disease onset was significantly lower in patients with no detectable drug levels, and the duration of Ifx treatment was significantly longer for patients with High drug levels. Sixteen patients in the no free Ifx group received an increased Ifx dose, and the treatment interval was reduced in eight patients. Eleven and nine patients in the low and high groups, respectively, received an increased Ifx dose, and the treatment interval was reduced in five and four patients, respectively. Both strategies were used simultaneously in some patients, and these numbers are higher. Five patients did not complete the year of treatment because of insufficient clinical response (three patients) and side effects (two patients, pneumonia and skin infection).

### Effectiveness of infliximab dose increase

**Figure 1:**
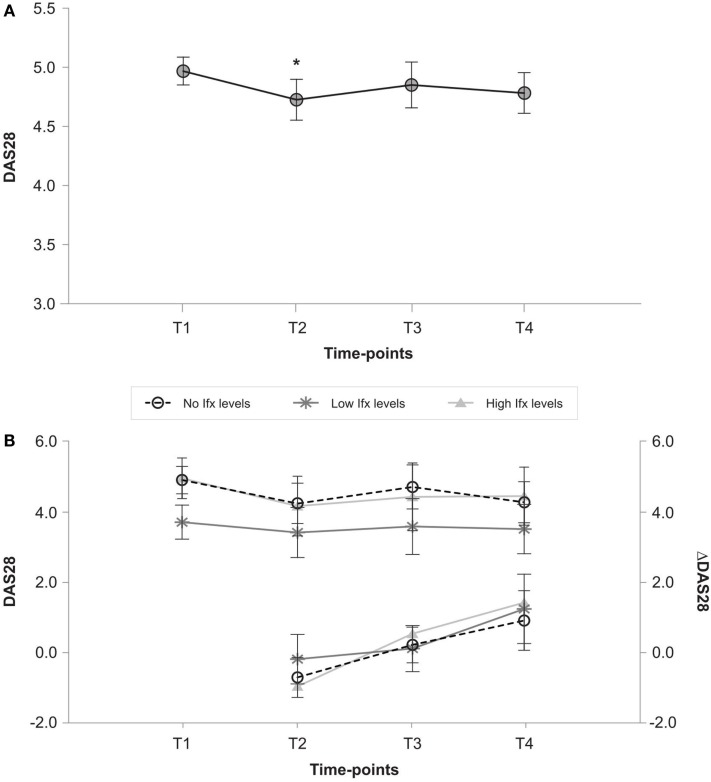
**(A)** Changes in DAS28 over time in all patients. T1 (baseline), T2 (post-Ifx dose increment), T3 (at 6 months), T4 (at 12 months). *p < 0.05 vs. DAS28 in T1 after Bonferroni correction. **(B)** DAS28 and delta-DAS28 from T1 in patients with no, low and high Ifx serum concentrations at baseline. T1 (baseline), T2 (post-Ifx dose increment), T3 (at 6 months), T4 (at 12 months).

Baseline DAS28 for the entire study population (Figure 1A) improved immediately after dose increase from baseline (4.55 ± 1.01 vs. 3.95 ± 1.22; *p* < 0.05), but this decrease in DAS28 disappeared at 12 months (3.98 ± 1.22; *p* = 0.075). The change in DAS28 from baseline (delta-DAS28) demonstrated significant disease worsening (from −0.63 ± 1.18 post-increase to 1.17 ± 1.45 after 12 months (*p* < 0.001). Figure 1B shows the DAS28 and delta-DAS28 progression for individual patient groups. Basal disease activity was lowest in the low group (3.7 ± 0.9) vs. 4.9 ± 0.7 (*p* = 0.001) and 4.9 ± 1.1 (*p* = 0.006) in the no detectable and high drug level groups, respectively). No significant change in DAS28 was observed in any of the individual patient groups throughout the study. The decrease in disease activity from the time of post-Ifx dose increase was significant in patients with no detectable Ifx levels after 12 months (mean delta-DAS28: 1.0 ± 1.9 vs. −0.7 ± 1.0, *p* < 0.05) and patients with high Ifx levels (mean delta-DAS28: 1.3 ± 1.3 vs. −1.0 ± 1.3; *p* < 0.05).

European League Against Rheumatism response rates in all three patient groups revealed no significant differences at any time point with 11.1, 16.7, and 12.5% of patients in the no, low, and high groups achieving a good response after the first dose increase, and 44.4, 58.3, and 37.5%, respectively, remained non-responders. Good responders after 12 months of enhanced treatment included 25% of no Ifx patients and 0% of low and high Ifx patients, and 50, 77.8, and 71.4% of no, low, and high patients, respectively, exhibited no response.

### Infliximab and anti-infliximab antibody concentrations after dose increase

**Figure 2:**
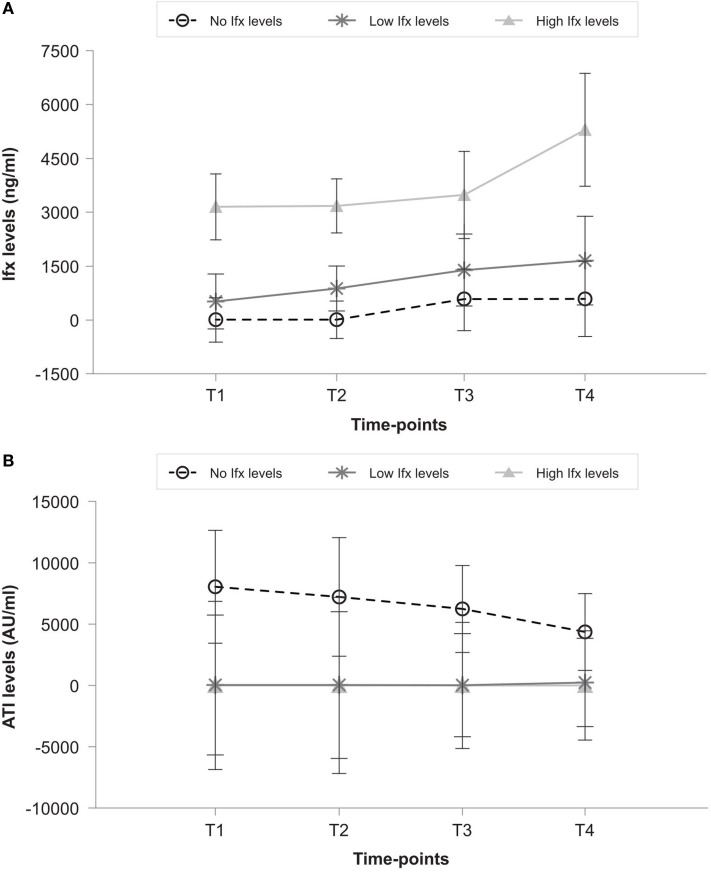
**(A)** Ifx levels and **(B)** ATI levels in patients with no, low, and high Ifx serum concentrations at baseline. T1 (baseline), T2 (post-Ifx dose increment), T3 (at 6 months), T4 (at 12 months).

Serum Ifx levels were significantly higher in the high group than the low and no groups at any studied points (Figure 2A). Ifx serum levels increased significantly between post-increment (T2) and 12 months (T4) in the high group (*p* = 0.017) but not in the low (*p* = 0.97) or no (*p* = 1) groups. No free Ifx was present at 12 months in 4 of 13 patients (30.7%) in the low group despite an increase in Ifx dose. Figure 2B shows that ATI levels in the no group ranged from a basal median of 1068.5 (IQR, 377.5–12328.0) AU/mL to 308.5 (IQR, 0.0–2805.0) AU/mL after 1 year, but it remained 0 AU/mL at all times in the low and high groups. ATI became positive with no free Ifx drug available in 3 of the 13 patients (23.0%) in the low group with previous negative ATI.

## Discussion

The therapeutic effect of an Ifx dose increase was analyzed in three populations of active non-remitting RA patients who exhibited no detectable, low and high drug levels. No significant improvement in disease activity or responder rate was observed after 1 year of intensified therapy, independently of pre-existing Ifx serum trough levels.

Our observations are consistent with anti-TNF failure treatment algorithms (7, 8) for two of the populations where a dose increase was ineffective, i.e., patients with high levels of Ifx and no response, and patients with non-detectable circulating Ifx due to the presence of ATI. However, treatment intensification is the recommended strategy in patients with low trough Ifx to achieve therapeutic levels of anti-TNF. Isolated analysis of this population did not reveal significant disease improvement despite dose increases in our study, and no significant increase in serum Ifx levels was achieved. Other studies increased the Ifx dose up to 10 mg/kg and found better results. Therefore, we do not conclude that the outcome would have been different with an increased Ifx dose up to 10 mg/kg. However, the cost to administer this dose is much higher (17).

Antibodies to infliximab were only detected in patients with no circulating Ifx because of drug interference with the method (5). The existence of ATI is highly improbable in patients with high Ifx levels, but the presence of hidden immunogenicity in the low Ifx level population may be partially responsible for low serum drug levels and poor outcomes. No Ifx was detected at 1 year in four patients, and three of these patients expressed ATI. Other non-immune Ifx clearance mechanisms, such as drug binding by immune cells expressing Fyc receptors I, II, and III, (18) naturally occurring anti-mouse antibodies binding infliximab (18, 19), and the “inflammation sink” in which highly expressing TNF tissues bind anti-TNF drug (18), may also contribute to the low circulating Ifx in these patients.

Our study has some limitations, such as the retrospective design, the lack of a pre-determined therapeutic protocol to increase Ifx dose, and the lack of a control group, but it reflects the effect of TNF-blocking agents that are used in daily clinical practice. The observational design of this study was also not appropriate for this type of analysis.

## Conclusion

The enhancing of Ifx therapy is costly and it poses the risk of increased adverse events (20), which stresses the importance of exploring Ifx efficacy. Increasing the dose of Ifx to counteract therapeutic response failure was unsuccessful in this study regardless of the circulating drug levels prior to dose escalation.

## Author Contributions

CP, DP-S, and AB were involved in the study design, patient selection and data collection and analysis. CP wrote the article. AV, LN, DP, and MB were directly involved in patient management. TJ, MC, and AM-F performed the serum assays and data collection. All authors were involved in the interpretation of the data and the critical review of the manuscript.

## Conflict of Interest Statement

Chamaida Plasencia has received research grants and speaker fees from Pfizer. Dora Pascual-Salcedo has received research grants and speaker fees from Pfizer, AbbVie, Novartis, and MSD. Alejandro Balsa and Emilio Martín-Mola have received unrestricted grants from Pfizer and AbbVie and speaker fees from Pfizer, Janssen, AbbVie, MSD, BMS, UCB, and Roche. The other authors have no competing interests for this research.
